# Influence of Alkali-Activators on Acid Rain Resistance of Geopolymer-Recycled Pervious Concrete with Optimal Pore Size

**DOI:** 10.3390/ma15238368

**Published:** 2022-11-24

**Authors:** Quan Ma, Wei Yang, Zhenhua Duan, Hui Liu, Minqi Hua, Qi Deng

**Affiliations:** 1 Department of Structural Engineering, Tongji University, Shanghai 200092, China; 2 Department of Civil Engineering, Changzhou University, Changzhou 213164, China; 3 School of Civil Engineering and Architecture, Wuhan University of Technology, Wuhan 430070, China

**Keywords:** geopolymer, pervious concrete, pore size, alkali-activators, acid rain

## Abstract

Geopolymer-recycled pervious concrete (GRPC) is a novel concrete that can effectively inhibit the corrosion of acid rain and alleviate urban waterlog. The goal of this study is to ascertain the optimal pore size of GRPC and study its acid rain resistance activated by different alkali-activators. Three different sizes (0.8, 1.0, and 1.2 mm) were separately chosen as the pore diameters of GRPC. The alkali-activator solution adopted sodium hydroxide (NaOH), sodium silicate (Na_2_SiO_3_), and a mixture of the two. The mechanical properties and permeability coefficient were tested to determine the optimal pore size of GRPC. After that, specimens with the optimal pore size were immersed in a simulative acid rain solution (sulfuric acid solution with pH = 4.0) for 6 d and were dried 1 d until 56 d. The effects of different alkali activators on acid rain resistance of GRPC were analyzed by compressive strength, neutralization depth, and mass loss. The results manifested that the mechanical properties of GRPC were excellent, the compressive strength of GRPC_H+N_ reached more than 60.1 MPa, and their splitting tensile strength attained more than 5.9 MPa, meeting the strength requirement of the road for heavy traffic load. Considering the mechanical properties and the acid rain purification effect of alkaline GRPC required a relatively small permeability coefficient; the optimal pore size was 1 mm. When specimens with optimal pore size were exposed to acid solution, the corrosion products (gypsums) would block the pores of GRPC to inhibit further corrosion, keeping the stability of the compressive strength. GRPC activated by the mixture of NaOH and Na_2_SiO_3_ generated a more stable amorphous three-dimensional network structure, endowing GRPC_H+N_ with better mechanical properties and acid corrosion resistance.

## 1. Introduction

In recent years, with the rapid development of the industry, the frequency, range and intensity of acid rain pollution have increased dramatically [1]. About 5.0% of China was reported to have suffered from acid rain attacks in 2019, covering an area of about 47.4 million hectares in the country [2]. It has been shown that the H^+^ from acid rain neutralizes the alkalinity of concrete, contributing to the pH of concrete decreasing and its internal volume expanding until failure [3,4]. According to estimates, acid rain attacks on concrete pavement in China result in annual economic losses of up to tens of billions of yuan [4]. In addition, the lack of water permeability in conventional concrete pavement significantly stops water on the road from discharging into underground water [5]. This can cause waterlogging in cities and threaten the safety of people’s lives and property. Thus, there are needs to develop a novel concrete with good resistance to acid rain attack and water permeability.

Geopolymer is a novel binding material, which is formed by the geopolymerization of an active aluminosilicate precursor (fly ash, metakaolin and ground granulated blast furnace slag, etc.) [6] and alkali activator [7,8]. In comparison with ordinary Portland cement (OPC), it not only possesses excellent engineering properties of high strength, high-temperature resistance, low permeability and acid corrosion resistance [9,10,11] but can also reduce carbon emissions by 20–50% [12,13]. Due to the high alkali content of geopolymer, its acid resistance is superior to cement. Thereby, the excellent acid resistance and high alkali content endow geopolymer with the potential of mitigating or eliminating acid rain corrosion [14]. With the continuous acceleration of urbanization, the output of construction and demolition wastes (C&DWs) is also increasing year by year. Recycling of C&DWs as recycled coarse aggregate to prepare recycled concrete (RAC) can effectively solve the problem of wastes and save the resource of natural aggregate [15]. Hence, the use of geopolymer as a binding material and recycled coarse aggregate (RA) as a coarse aggregate to prepare GRPC is a meaningful attempt.

Pervious pavement concrete plays a vital role in the urban rainwater management system. Knowing the permeability penetration rate for a pervious pavement system, which can successfully minimize the runoff volume, is significant to comprehend how pervious concrete pavement drains water [16]. Concerning pervious concrete, many scholars have performed research. Li et al. [5] utilized reactive powder concrete as the matrix to prepare straight-through pore-pervious concrete, which effectively improved its low strength and high likelihood of clogging of conventional pervious concrete pavement. Prinya et al. [17] made use of the alkaline properties of the geopolymer, successfully preparing the pervious geopolymer concrete to neutralize acidic wastewater from the operational processes of a power plant. Vanchai et al. [18] used two different types of RA, viz., crushed structural concrete member (RC) and crushed clay brick (RB), to make GRPC. The results indicated that both RC and RB can be used as RA for making GRPC with acceptable properties. Lu et al. [19] prepared an eco-friendly pervious concrete with waste glass cullet (WGC), and they found that the concrete comprising 50% WGC as the fine aggregate and 50% RA as the coarse aggregate could achieve satisfactory strength and permeability. However, they only studied the pervious concrete with a single pore size and the feasibility of using RA in geopolymer pervious concrete. Systematic research on the impact of GRPC’s pore size is still lacking.

Concrete durability is the ability of concrete to withstand the effects of harsh environmental media that jeopardize the regular operation of concrete components [9]. Acid rain attack is one of the most typical ways that threaten concrete’s service life. In studies thus far, a great deal of work has been put into the acid resistance of geopolymer materials by researchers. Yang et al. [20] studied the sulfuric acid resistance of geopolymer concrete with different binding materials and concentrations of NaOH. They concluded that the formation of gel depended on the concentration of alkali OH ion, high NaOH concentration endowed concrete with better compressive strength and acid resistance. Bakharev [21] investigated the durability of three geopolymer materials activated by Na_2_SiO_3_, NaOH and a mixture of sodium and potassium hydroxides exposed to an acid environment, and they manifested a geopolymer material activated by NaOH with the best performance due to its more stable cross-linked aluminosilicate polymer structure. Although researchers have performed many studies on the effect of alkali-activators on geopolymer material, few of them take note of the influence of alkali-activators on geopolymer pervious concrete.

The work reported herein is aimed to determine the optimal pore size of GRPC and study its acid rain resistance activated by different alkali-activators. Three different sizes (0.8 mm, 1.0 mm, and 1.2 mm) were selected as the pore diameters of GRPC. Compressive strength, splitting tensile strength, and permeability coefficient were tested to evaluate the permeability performance of specimens. Then, the optimal pore size of specimens was determined. On this basis, the acid rain resistance of specimens activated by NaOH, Na_2_SiO_3_, and the mixture of the two was studied. Compressive strength, neutralization depth and mass loss were adopted to analyze the acid rain resistance of GRPC.

## 2. Materials and Methods

### 2.1. Raw Materials

**Figure 1:**
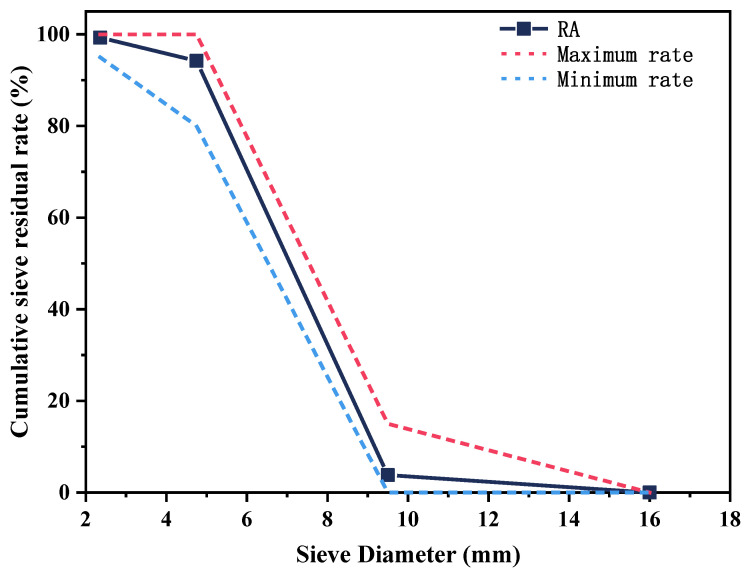
Granulometric curve of recycled coarse aggregate.

The high calcium fly ash (FA) with the density of 2500 kg/m^3^ from Changzhou Hutang Thermoelectric Co., Ltd., Changzhou, China, and its chemical composition were measured by X-ray fluorescence (XRF), as shown in Table 1. The recycled coarse aggregate (RA) was provided by Jiangsu Lvhe Environmental Technology Co., Ltd., Changzhou, China. The particle size of RA was adopted at 5–10 mm, which was in compliance with Chinese Standard GB/T 25177-2010 [22], to produce the freshly mixed GRPC with appropriate workability and to enable the smooth creation of pervious concrete pores. Its granulometric curve is shown in Figure 1. Washed river sand with the fineness modulus of 2.5 was fine aggregate. The reagents of sodium hydroxide (NaOH ≥ 96.0%, AR), sulfuric acid (H_2_SO_4_ ≥ 95.0%, AR) and phenolphthalein (1%) were purchased from Sinopharm Chemical Reagent Co., Ltd., Shanghai, China. Sodium silicate liquid (Na_2_SiO_3_ with 29.9 wt% SiO_2_, 13.75 wt% Na_2_O, and 56.35 wt% H_2_O) and distilled water were used in the experiments.

### 2.2. Mix Proportions and Preparation of Specimens

Not all of the alumina in the fly ash geopolymerization system participates in the geopolymerization reaction, nor is it necessary for a Na ion to balance every negative charge on the silica–alumina tetrahedra [23]. An appropriate decrease in the Na_2_O/Al_2_O_3_ ratio is beneficial to the geopolymerization reaction and will improve the strength of the geopolymer. Therefore, in this study, the Na_2_O/Al_2_O_3_ ratio was taken as 0.7, while Na_2_O/H_2_O = 1:12 was selected. Fly ash and alkali-activator solution were mixed according to the mixing proportion given in Table 2. The alkali-activator solution consisted of Na_2_SiO_3_, NaOH (12 M), and the mixture of the two. The NaOH solution should be prepared 24 h in advance.

**Figure 2:**
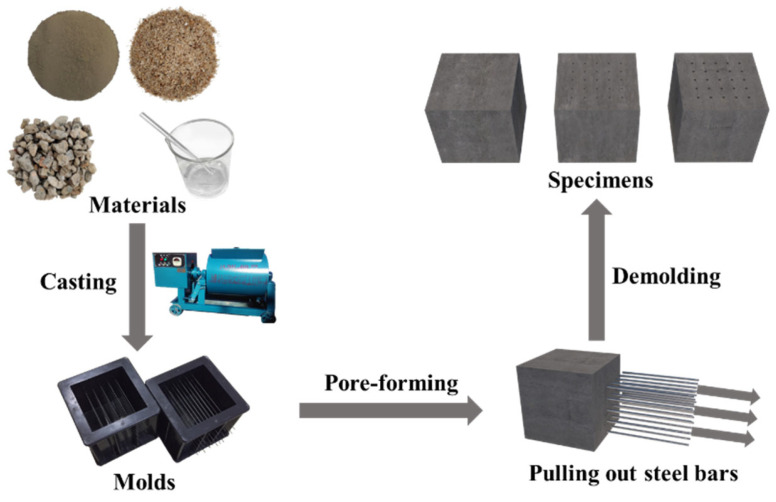
Preparation process of GRPC.

The preparation process of GRPC is shown in Figure 2. All molds were pre-inserted with steel bars, whose diameters were 0.8, 1.0 and 1.2 mm. The layout of the straight-through pore was the array of 5 × 5, and the number of steel bars was 25 in total. Then, the mixtures were poured into the molds of 100 × 100 × 100 mm^3^. The mixtures needed to be filled in three layers. After filling, the specimens were covered with plastic film to prevent the evaporation of water and stood for 24 h at room temperature. Next, the steel bars were pulled out and specimens were put into hermetic bags, curing at 60 °C for 48 h. After that, specimens cooled for 1 h were demolded and preserved in the standard curing room (20 ± 2 °C, RH ≥ 95%) until the age of 28 d. The porosities of the specimens with pore sizes of 0.8, 1.0, and 1.2 mm were 0.13%, 0.20%, and 0.28%, respectively.

### 2.3. Measurements

#### 2.3.1. Mechanical Properties

Compressive strength and splitting tensile strength of GRPC specimens with different alkali-activators and pore sizes were tested by electro-hydraulic servo universal testing machine (YNS 300) according to Chinese Standard GB/T 50081-2019 [24]. Both the compressive strength and splitting tensile strength were tested parallel to the pores. The value of compressive strength and splitting tensile strength were the average of three specimens.

#### 2.3.2. Permeability Coefficient

The permeability coefficient of GRPC was tested according to Chinese Standard CJJ/T 135-2009 [25]. The constant head method was adopted to determine the permeability coefficient. The calculation formula is as follows:

(1) $$K=\frac{\mathrm{QL}}{\mathrm{AHt}}$$

where *K* is the permeability coefficient (mm/s), Q is the volume of water flowing out of the spillway in t seconds (m^3^), L is the height of the specimen (mm), A is the surface area of the top surface of the specimen (m^2^), H is water level difference (m), and t is the time of water flowing. In this experiment, L was the height of the specimen of 100 mm, the water level difference was 150 mm, and the value of t was 30 s. The experimental results were the average of three specimens.

#### 2.3.3. Acid Rain Resistance Test

The specimens were immersed in the sulfuric acid solution with pH = 4 for 6 days, and then taken out to dry for 1 day. The whole cycle lasted for 56 days. Furthermore, the concentration of acid solution was renewed every week to maintain a stable pH value. Compressive strength, neutralization depth and mass loss were separately used to characterize the acid rain resistance of GRPC.

After all specimens were immersed in the acid solution for 7, 14, 28, and 56 days, the neutralization depth of specimens was tested by spraying 1% phenolphthalein indicator on the cross-sections of the GRPC pores. The distance between the edge of the specimen and the discoloration boundary was measured by a vernier caliper. Eight points on each section were selected for measurement, and the arithmetic mean value was taken as the neutralization depth.

The mass loss of specimens exposed to acid solution for 7, 14, 28, and 56 days was calculated by the formula:

(2) $$W=\frac{m_{0}-m_{1}}{m_{0}}\times 100\%$$

where W is the mass loss rate; *m*_0_, and *m*_1_ are the masses of specimens before and after soaking in sulfuric acid, respectively, in g. The time points of measurement were 0, 7, 14, 28, and 56 days.

## 3. Results and Discussion

### 3.1. Mechanical Properties and Water Permeability of GRPC

#### 3.1.1. Mechanical Properties

**Figure 3:**
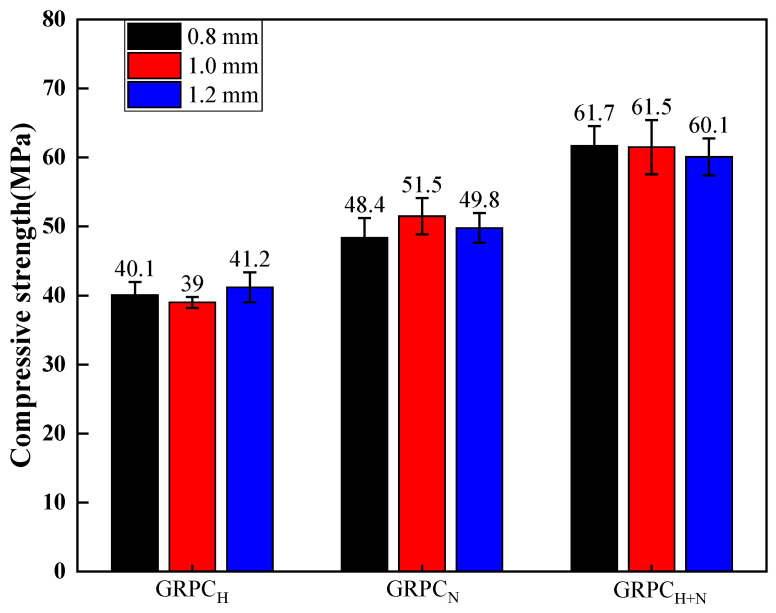
Compressive strength of GRPC with different alkali-activators and pore sizes.

Figure 3 reveals the compressive strength of GRPC with different alkali-activators and pore sizes at 28 d. As we can see from Figure 3, the compressive strength of GRPC was relatively high. Except for GRPC_H_ with the pore size of 1.0 mm, the compressive strength of other specimens was over 40 MPa, which met the requirements of light- and medium-traffic road load-bearing capacities [26]. Meanwhile, the compressive strength of GRPC_N_ with the pore size of 1.0 mm and GRPC_H+N_ with three different pore sizes reached more than 50 MPa, meeting the strength requirement of the road for heavy traffic load [26]. The porosities of GRPC with three different pore sizes (0.8, 1.0, 1.2 mm) were 0.13%, 0.20% and 0.28%, respectively. It was precise because of the low porosity of GRPC, where the compressive strength of concrete remained high. In addition, due to the pore size of GRPC being relatively small, the compressive strength of GRPC under the action of the same alkali-activator was less affected by pore size. The main factor that caused significant differences in the compressive strength of GRPC was the type of alkali activators. Due to the quicker geopolymerization reaction between the sources and alkaline solutions, specimens activated by the combination of NaOH and Na_2_SiO_3_ had the maximum compressive strength. When compared with the conventional pervious concrete (with large voids and poor bond strength of the mortars) [27], the compressive strength of GRPC improved significantly.

**Figure 4:**
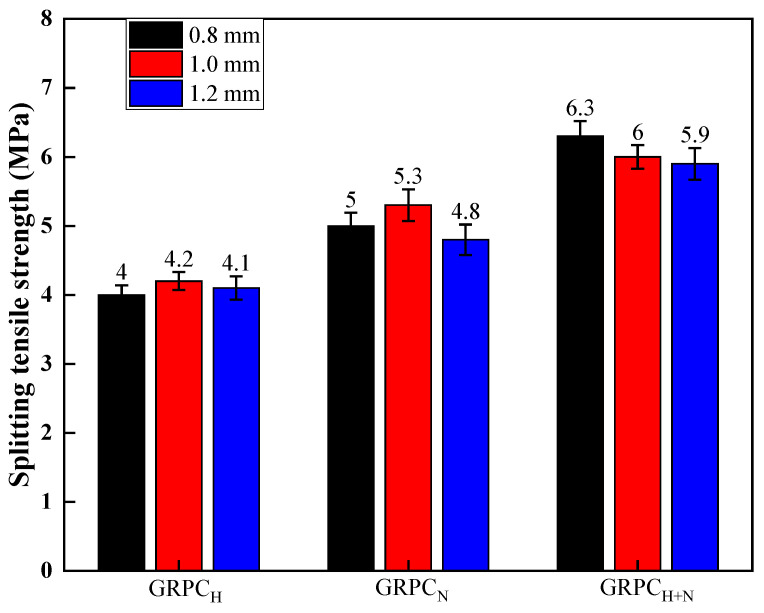
Splitting tensile strength of GRPC with different alkali-activators and pore sizes.

The splitting tensile strength of GRPC with different alkali-activators and pore sizes at 28 d is shown in Figure 4. The specimens activated by NaOH had the lowest splitting tensile strength, the values of GRPC (with pore sizes of 0.8, 1.0, and 1.2 mm) were 4, 4.2, and 4.1 MPa, respectively. On the contrary, the strength of specimens activated by the mixture of NaOH and Na_2_SiO_3_ was the highest, and the splitting tensile strength of GRPC with the pore size of 0.8, 1.0, and 1.2 mm was 6.3, 6, and 5.9 MPa. It was attributed to a more stable cross-linked aluminosilicate polymer structure formed in GRPC_H+N_ [28]. Furthermore, it can be found that the splitting tensile strength of the specimens was activated by the same alkali-activator without much difference. The pore size had little effect on the splitting tensile strength of GRPC, which was consistent with the results found in the compressive strength test. Thus, it can be concluded that the mechanical properties of GRPC are mainly determined by the properties of its matrix in the case of small pore size. However, when the pore size of GRPC was 1.2 mm, the splitting tensile strength was the lowest among the three pore sizes. This was due to the existence of straight-through pores in GRPC, and the tensile surface turned weaker as the pore diameter enlarged. Thus, as the pore size of GRPC expanded, the splitting tensile strength was obviously weakened.

#### 3.1.2. Water Permeability

**Figure 5:**
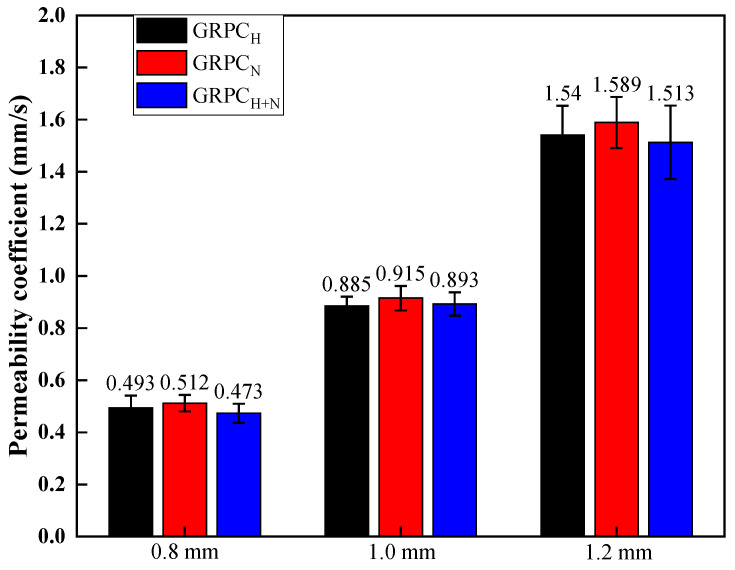
Permeability coefficient of GRPC with different alkali-activators and pore sizes.

**Figure 6:**
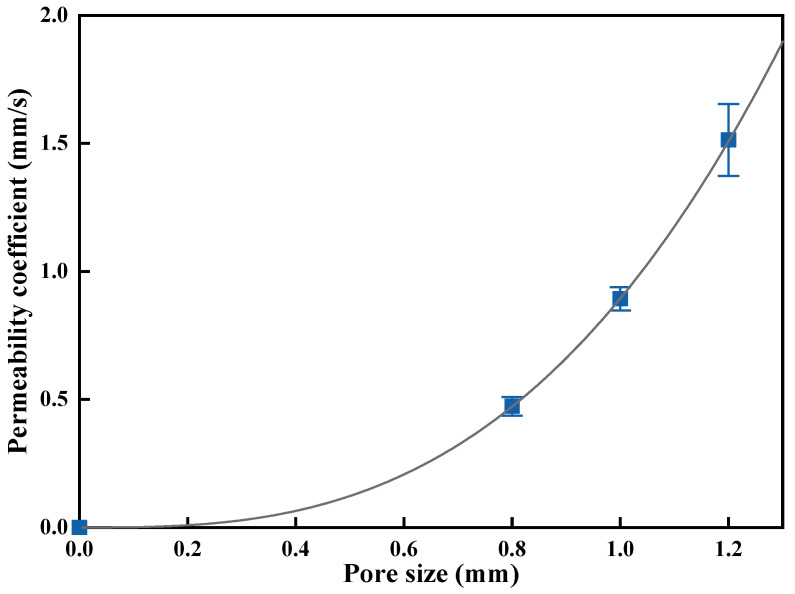
The relationship between permeability coefficient and GRPC_H+N_ pore size.

The permeability coefficient is the key parameter to evaluate the water permeability of GRPC [29]. Figure 5 presents the permeability coefficient of GRPC with three different pore sizes. When the pore size was 0.8 mm, the permeability coefficients of GRPC_N_ and GRPC_H+N_ were 0.493 and 0.473 mm/s, respectively, which did not meet the requirements specified [25]. However, the permeability coefficients of other specimens were more than 0.5 mm/s, conforming to the requirements of specification. With the pore size of GRPC enlarged, the permeability coefficient increased correspondingly. Figure 6 reveals the relationship between permeability coefficient and pore size of GRPC_H+N_. There is a good correlation between permeability coefficient and pore size, and they are related in a power function. The functional relation is as follows:

(3) $$y=0.895\times x^{2.863}{\;R}^{2}=0.99$$

where y is the permeability coefficient, and x is the pore size. The R^2^ is 0.99, suggesting a good correlation between permeability coefficient and pore size.

If the smaller pore size was taken, the pore of GRPC would appear blocked and affect the permeability coefficient of GRPC. Thereby, the specimens’ function of permeability, purifying acid rain, etc., weakened accordingly [30]. Thus, the 0.8 mm was not suitable for the optional pore size of GRPC. When the pore sizes of 1.0 and 1.2 mm were adopted, the average permeability coefficients were 0.9 and 1.55 mm/s, respectively, which met the requirements of specification. At the flow rate of 0.9 mm/s, the acid rain passed through the pores of the specimens slowly, providing sufficient time for the reaction between GRPC and acid rain to ensure the purification effect of GRPC. Considering both mechanical and permeability properties, the optimal pore size of GRPC was 1.0 mm.

### 3.2. Acid Rain Resistance of GRPC

#### 3.2.1. Compressive Strength

**Figure 7:**
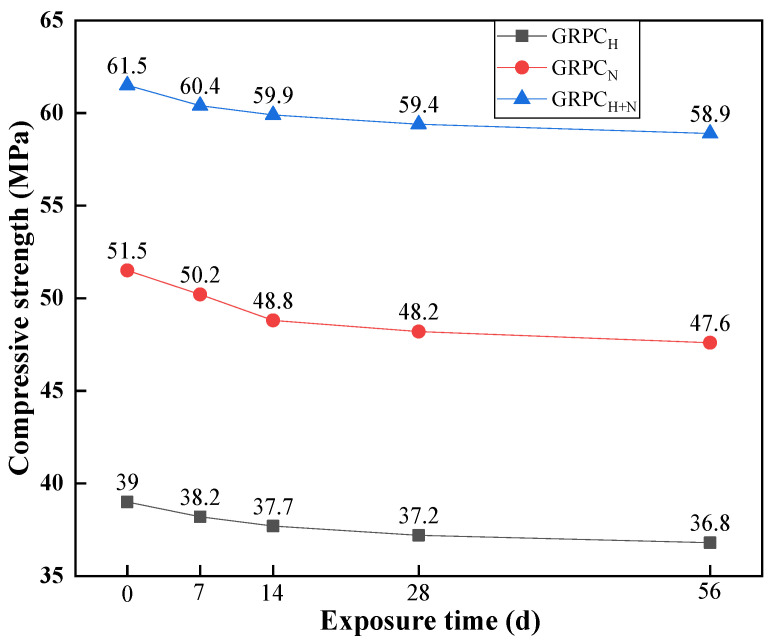
Compressive strength of specimens at different exposure times.

Figure 7 renders the evolution of compressive strength of GRPC with optimal pore size (1.0 mm) exposed to sulfuric acid solution for 0, 7, 14, 28, and 56 d. As can be seen from the figure, when specimens were unexposed to sulfuric acid solution, the compressive strength of GRPC_H_, GRPC_N_, and GRPC_H+N_ was 39, 51.5 and 61.5 MPa. The compressive strength of GRPC activated by the mixture of NaOH and Na_2_SiO_3_ was evidently higher than the other ones. It can be explained that this kind of alkali-activator can promote the geopolymerization of geopolymer and accelerate the formation of gel phase (N-A-S-H). Catherine et al. [31] proved this result by the infrared in situ monitoring method.

After the erosion of sulfuric acid solution, the compressive strength of GRPC declined with the increase in exposure time. When specimens were immersed in sulfuric acid solution for 14 d, the compressive strengths of GRPC dropped at a relatively rapid rate. The falling speeds of GRPC_H_, GRPC_N_, GRPC_H+N_ were 2.6%, 5.2%, 3.3%, respectively. It was the pores of specimens that absorbed sulfuric acid solution and reacted with RA that resulted in the decrease in compressive strength. After that, the compressive strength of GRPC declined slowly, which was attributed to the calcium sources in specimens reacting with SO_4_^2−^ to produce the expansive products of gypsums, filling in the pores of concrete and inhibiting further corrosion [32].

When the exposure time was up to 56 d, the compressive strengths of GRPC_H_, GRPC_N_, and GRPC_H+N_ were 36.8, 47.6, and 58.9 MPa, respectively. Among them, the compressive strength of GRPC_H+N_ still met the requirement of roads for heavy traffic loads [26]. It was due to the fact that the hydrated sodium aluminosilicate product (N-A-S-H gel) generated by the geopolymerization reaction had a strong bond, which can maintain relative chemical stability in acidic media with low concentrations (pH ≥ 4.0) [33]. By contrastive analysis, it can be found that GRPC activated by the mixture of NaOH and Na_2_SiO_3_ generated the more stable amorphous three-dimensional network structure, endowing GRPC_H+N_ with better mechanical properties and acid corrosion resistance [34]. Hence, the mixture of NaOH and Na_2_SiO_3_ was the optimal alkali-activator to prepare GRPC.

#### 3.2.2. Neutralization Depth

**Figure 8:**
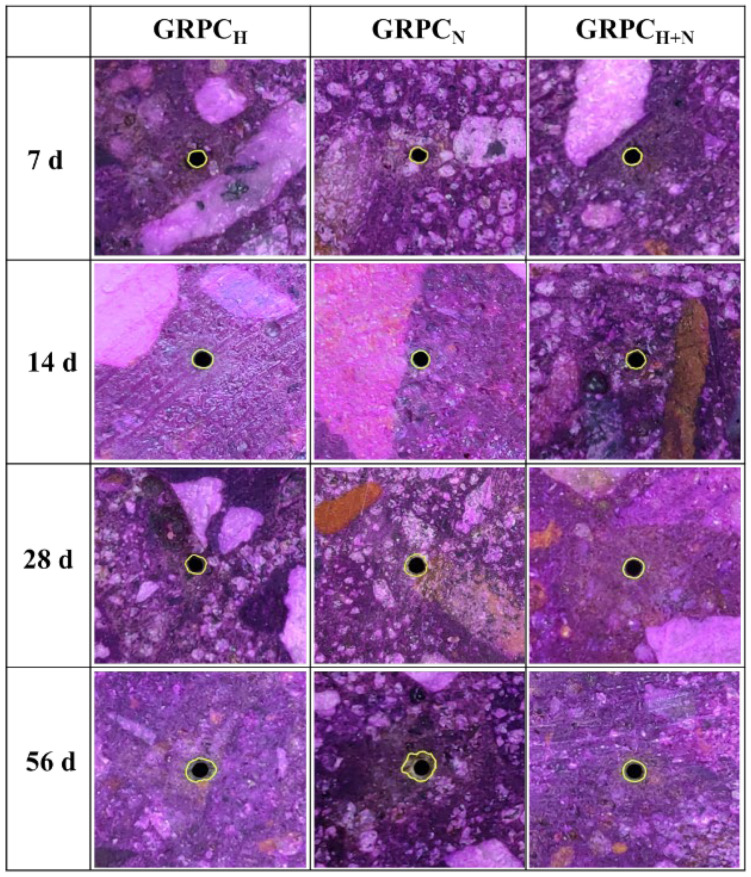
Variation of cross-section of GRPC pores.

The neutralization depth or ion exchange area of the specimen is related to the alkalinity loss [35]. Spraying phenolphthalein solution on the surface of the concrete’s cross-section can contribute to its section turning fuchsia, displaying the penetration depth of the acid [36]. Cross-sections of GRPC pores exposed to acid solution for 7, 14, 28 and 56 d are shown in Figure 8. As can be seen from the figure, when GRPC was exposed to sulfuric acid solution with pH = 4.0 for 56 d, the appearance of GRPC did not change significantly, and the cracking or breakage did not appear on the surface of the specimens. Furthermore, the residual alkalinity of all GRPC sections was still high, and the whole sections were nearly the color of fuchsia. However, the slightly irregular neutralization depth interface was observed in GRPC_N_, marking the occurrence of corrosion.

**Figure 9:**
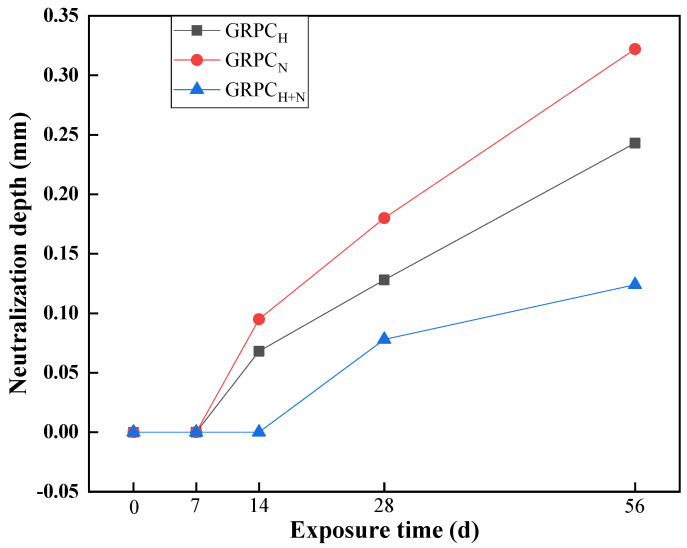
Neutralization depth of specimens at different exposure times.

The neutralization depth of GRPC with optimal pore size (1.0 mm) exposed to sulfuric acid solution for 7, 14, 28, and 56 d is shown in Figure 9. It can be observed that the neutralization depth of GRPC increased along with the extension of exposure time. At the early stage, the neutralization depth of three different GRPC specimens did not make changes. When the exposure time was 14 d, the neutralization depth of GRPC_H_ and GRPC_N_ increased to 0.068 and 0.095 mm. However, the neutralization depth of GRPC_H+N_ was kept intact. At the exposure time of 56 d, the neutralization depths of GRPC_H_, GRPC_N_ and GRPC_H+N_ were only 0.243, 0.322 and 0.124 mm. The superior performance of GRPC was attributed to its stable cross-linked structure compared with ordinary Portland cement concrete [37]. Furthermore, it was found that the GRPC activated by the mixture of NaOH and Na_2_SiO_3_ was more insensitive to acid solution than other specimens. It can be explained that the geopolymerization of the geopolymer material activated by the mixture of NaOH and Na_2_SiO_3_ was more complete, and the stable hydration product (N-A-S-H gel [38]) strengthened the acid resistance of GRPC.

#### 3.2.3. Mass Loss

**Figure 10:**
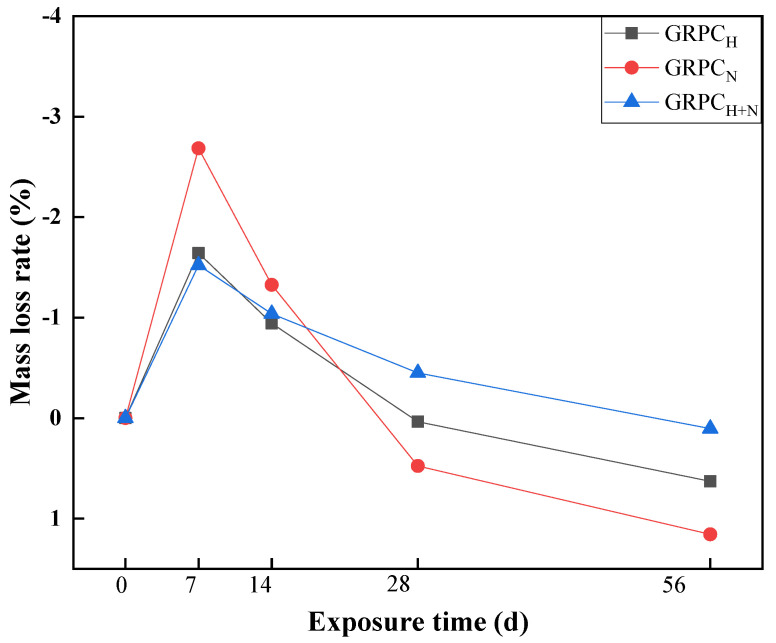
Mass loss rate of specimens at different exposure times.

Specimens exposed to acid erosion medium for a long time; their mortars would peel off from the matrix and contribute to the mass loss of concrete. The main reason for the mass loss of the geopolymer material was the depolymerization of aluminosilicate gel and the leaching of ions from the matrix [39]. Figure 10 presents the mass loss rate of GRPC with optimal pore size (1.0 mm) exposed to sulfuric acid solution for 7, 14, 28, and 56 d. As can be seen from the figure, with the increase in exposure time, the mass of GRPC showed a general trend of increasing and then decreasing. At the early stage, the pores of GRPC absorbed acid solution and reacted with the RA connected to the pores, resulting in the gypsum accumulating in the pores of the GRPC matrix and increasing the mass of GRPC, which was consistent with previous reports [40,41,42].

When specimens were exposed to sulfuric acid solution for 14 d, the mass of GRPC started to decline. At the exposure time of 56 d, the mass loss rates of GRPC_H_, GRPC_N_, GRPC_H+N_ were 0.63%, 1.16%, 0.10%, respectively. The mass loss rate of GRPC was small, which can be attributed to the cross-linked N-A-S-H gel generated in the matrix of the geopolymer, which was structurally stable under weak acid conditions [43]. In addition, it can be found that the mass loss rate of GRPC_H+N_ was less than the other two specimens, which can be explained by the denser structure of GRPC_H+N_, which endowed the specimen with better acid resistance.

## 4. Conclusions

The influence of alkali-activators on acid rain resistance of geopolymer-recycled pervious concrete with the optimal pore size was studied in this paper. Based on the experimental and analytical results, the main conclusions can be summarized as follows:

(1) The mechanical properties of GRPC were excellent, except for GRPC_H_ with the pore size of 1.0 mm. The compressive strength of other specimens surpassed 40 MPa and the splitting tensile strength exceeded 4 MPa, meeting the requirements of light and medium traffic roads’ load-bearing capacity. The compressive strength of GRPC_H+N_ reached more than 60.1 MPa, and the splitting tensile strength attained more than 5.9 MPa, meeting the strength requirement of a road for heavy traffic load.
(2) The permeability coefficient depended mainly on the pore size and had little correlation with the matrix properties. The average permeability coefficients of GRPC with pore sizes of 0.8, 1, and 1.2 mm were 0.5, 0.9, and 1.55 mm/s. Considering that the pore size of 0.8 mm was prone to clogging, which affected the permeability of GRPC, the smaller permeability coefficient had the better effect on acid rain purification. Therefore, the optimal pore size of GRPC was 1 mm.
(3) When specimens were exposed to acid solution, the pores of GRPC absorbed the acid solution and reacted with some of the RA connected with the pores, resulting in a reduction of compressive strength. However, the gypsum generated by the reaction blocked the pores to inhibit further corrosion, keeping the stability of the compressive strength.
(4) The GRPC activated by the mixture of NaOH and Na_2_SiO_3_ generated the more stable amorphous three-dimensional network structure, endowing GRPC_H+N_ with better mechanical properties and acid corrosion resistance.

## Figures and Tables

**Table 1:** Chemical compositions of fly ash.

Components	SiO_2_	Al_2_O_3_	Fe_2_O_3_	TiO_2_	CaO	K_2_O	MgO
Weight (%)	44.18	26.92	9.34	1.34	11.02	1.39	1.88

**Table 2:** Mix proportions of GRPC (kg/m^3^).

Mixes	Fly Ash	Recycled Coarse Aggregate	Sand	Na_2_SiO_3_	NaOH	Water
GRPC_H_	541.3	912.7	559.4	-	80	198
GRPC_N_	541.3	655.4	401.7	476.2	-	58.9
GRPC_H+N_	541.3	784.1	480.6	238.1	40	128.4

Notes: GRPC_H_, GRPC_N_, GRPC_H+N_ indicate that the alkali-activator solutions are NaOH, Na_2_SiO_3_, NaOH + Na_2_SiO_3_, respectively.

## Data Availability

Not applicable.
